# Development and Characterization of Recombinant Antibody Fragments That Recognize and Neutralize *In Vitro* Stx2 Toxin from Shiga Toxin-Producing *Escherichia coli*

**DOI:** 10.1371/journal.pone.0120481

**Published:** 2015-03-19

**Authors:** Daniela Luz, Gang Chen, Andrea Q. Maranhão, Leticia B. Rocha, Sachdev Sidhu, Roxane M. F. Piazza

**Affiliations:** 1 Laboratório de Bacteriologia, Instituto Butantan, São Paulo, Brazil; 2 Banting and Best Department of Medical Research, Terrence Donnelly Centre for Cellular and Biomolecular Research, University of Toronto, Toronto, Canada; 3 Laboratório de Imunologia, Universidade de Brasília, Brasília, Brazil; USDA-ARS-ERRC, UNITED STATES

## Abstract

**Background:**

Stx toxin is a member of the AB_5_ family of bacterial toxins: the active A subunit has N-glycosidase activity against 28S rRNA, resulting in inhibition of protein synthesis in eukaryotic cells, and the pentamer ligand B subunits (StxB) bind to globotria(tetra)osylceramide receptors (Gb3/Gb4) on the cell membrane. Shiga toxin-producing *Escherichia coli* strains (STEC) may produce Stx1 and/or Stx2 and variants. Strains carrying Stx2 are considered more virulent and related to the majority of outbreaks, besides being usually associated with hemolytic uremic syndrome in humans. The development of tools for the detection and/or neutralization of these toxins is a turning point for early diagnosis and therapeutics. Antibodies are an excellent paradigm for the design of high-affinity, protein-based binding reagents used for these purposes.

**Methods and Findings:**

In this work, we developed two recombinant antibodies; scFv fragments from mouse hybridomas and Fab fragments by phage display technology using a human synthetic antibody library. Both fragments showed high binding affinity to Stx2, and they were able to bind specifically to the GKIEFSKYNEDDTF region of the Stx2 B subunit and to neutralize *in vitro* the cytotoxicity of the toxin up to 80%. Furthermore, the scFv fragments showed 79% sensitivity and 100% specificity in detecting STEC strains by ELISA.

**Conclusion:**

In this work, we developed and characterized two recombinant antibodies against Stx2, as promising tools to be used in diagnosis or therapeutic approaches against STEC, and for the first time, we showed a human monovalent molecule, produced in bacteria, able to neutralize the cytotoxicity of Stx2 *in vitro*.

## Introduction

Shiga toxin (Stx)-producing *Escherichia coli* (STEC) are bacterial pathogens responsible for a spectrum of diseases, ranging from asymptomatic carriage (rare) to diarrhea, bloody diarrhea, hemorrhagic colitis (HC) and hemolytic uremic syndrome (HUS) [1]. STEC strains are known to carry inducible lambda phages integrated into their genomes, which encode Stx toxins and can exist as two different types and their variants, including three Stx1 (Stx1a, Stx1c and Stx1d) and seven Stx2 (from Stx2a to Stx2g) subtypes. Stx1a and Stx2a are the prototypes for these toxins [2, 3]. These phages can be easily exchanged through horizontal gene transfer [4]. The Stx2 and Stx2c toxins are considered more virulent and epidemiologically most related to outbreaks [5, 6], besides being usually related to HUS in humans [7]. Stx toxins are members of the AB_5_ family of bacterial toxins, in which the pentamer ligand B subunits (StxB) bind to globotria(tetra)osylceramide receptors (Gb3/Gb4) on the cell membrane and translocate the active A subunit (StxA), which possesses N-glycosidase activity against 28S rRNA of 60S ribosomes into the cytosol, resulting in inhibition of protein synthesis in eukaryotic cells [8,9].

Currently, two different aspects deserve attention regarding this pathogen, early diagnosis (based on the patient and the source of the outbreak) and the therapeutic approach. Routine laboratory diagnoses of STEC strains are based on isolation from stool specimens [10], detection of Stx in fecal filtrates [11] and/or antibody-based methods against Stxs [3,12,13,14,15,16,17]. Moreover, these tests basically focus on the screening for the O157:H7 serotype, the most outbreak-related serotype, even though lately, other serotypes have emerged as food poisoning agents, such as O104:H4, which caused a major pathogenic *E*. *coli* outbreak that occurred in central Europe in 2011 [9].

Regarding intoxication treatment, antibiotics are not recommended for STEC infections, since Stxs are encoded by phages, whose expression is driven by cellular stress, so antibiotic therapy would induce the SOS response, which could increase the level of Stx delivery [3]. Presently, treatment is limited to fluid replacement and supportive care. One alternative treatment for STEC infection and possibly for HUS is neutralizing anti-Stx antibody therapy. Monoclonal antibodies (mAb) against Stx have been evaluated in animal models [18,19,20,21,22,23,24]. One in particular, urtoxazumab showed better prospects in HUS therapy, as it appears to be a safe therapeutic tool [24]. Nonetheless, it remains unknown whether antitoxin antibodies administered after the onset of diarrheal symptoms will prevent or modify the outcome of HUS. Even if effective, generating monoclonal antibodies is an expensive and time-consuming process [25].

Innovative recombinant DNA technologies, including chimerization and humanization, have enhanced the clinical efficacy of murine mAb and, in the past decade, have led to regulatory approvals for immunoglobulin (Ig) and classic monovalent antibody fragment (Fab) molecules, either for therapy or diagnostic tools [25]. Furthermore, recombinant antibodies (rAbs) have been dissected into minimal binding fragments such as scFv rebuilt into multivalent high-avidity reagents used for various purposes [26]. Some recombinant antibodies against Stx2 were developed and shown to be functional; however these are not yet commercially available for either therapy or diagnosis [23,27,28].

Taking into consideration the importance of STEC infections and the intoxication with Stx toxins, in addition to the urgency for faster and easier detection of these strains in sources of foodborne outbreaks, we made an effort to isolate and produce two different engineered antibodies against Stx2 toxin from Shiga toxin-producing *Escherichia coli*. Considering their use as tools for diagnosis and treatment, we used the following approaches: scFv isolation from a previously characterized hybridoma and Fab selection from a human synthetic phage library by phage display technology.

## Materials and Methods

### Ethics statement

No animal model was employed in the present work. The hybridoma used as template for scFv development was obtained according to Rocha et al. [14], and as described, the experiments were conducted in agreement with the Ethical Principles in Animal Research, adopted by the Brazilian College of Animal Experimentation, and they were approved by the Ethical Committee for Animal Research of Butantan Institute (469/08). Vero cells used in the neutralization assays are from African green monkey kidney (ATCCCCL-81).

### Toxins, chemicals, reagents and supplies

Purified Stx1 and Stx2 were purchased from Tufts University School of Medicine, Boston, MA, USA. rProtein A Sepharose Fast Flow was bought from GE Healthcare, UK. Ni-NTA beads were purchased from Qiagen (Hilden, Germany). The enzymes used for cloning (*Nsi*I/*Sal*I and *Eag*I/*Sal*l) were bought from Thermo Scientific (MA, USA) and the T4 ligase enzyme was obtained from Invitrogen (CA, USA). HRP/anti-FLAG horseradish peroxidase conjugate and 3’3’-diaminobenzidine (DAB) were acquired from Sigma Aldrich (St Louis, MO, USA). Dulbecco’s medium (DMEM) and fetal bovine serum (FBS) were acquired from GibcoBRL (São Paulo, Brazil). For induction we used IPTG (isopropyl β-D-1-thiogalactopyranoside) from Thermo Scientific. For ELISA assays, we employed 96-wells or 384-well MaxiSorp microplates from Nunc (Rochester, NY, USA); the assays were developed with TMB substrate solution (3,3’,5,5’—tetramethylbenzidine) from Thermo Scientific, and absorbance was determined in a Multiskan EX ELISA reader from Labsystems (Milford, MA, USA).

### Bacterial strains and plasmids

*Escherichia coli* strains used were DH5a [*fhuA2 lac(del)U169 phoA glnV44 Φ80' lacZ(del)M15 gyrA96 recA1 relA1 endA1 thi-1 hsdR17*], BL21 (DE3) [*E*. *coli B dcm ompT hsdS*(r_B_^-^m_B_^-^) *gal*], Phage-resistant Omnimax 2 T1^R^ (Invitrogen, CA, USA), and BL21(DE3) pLysS (DE3) [*omp*T, *hsd*SB (rB–, mB–), *dcm*, *gal*, λ(DE3), pLysS, Cmr]. The plasmid vectors used were pFabHis-MBP and pscFvHIS-MBP for expression of recombinant Fab and scFv fragments in bacteria, respectively, as well as phagemid HP153 for Fab display on phage particles [29]. Plasmids pFabHis-MBP and pscFvHIS-MBP were constructed on the basis of vector Stop4 [30] by following standard molecular cloning protocol. The bacterial isolates used in this study were strains previously defined as STEC by gene presence and Stx1 or Stx2 production [14], including the prototype EDL933 (O157:H7) [31]. We also included for ELISA cut-off definition, 7 *E*. *coli* strains negative for diarrheagenic *E*. *coli* virulence factors, 16 diarrheagenic *E*. *coli* strains other than STEC, and 20 Enterobacteriaceae isolates (*Edwardsiella tarda*, *Enterobacter cloacae*, *Klebsiella* spp., *Proteus mirabilis*, *Providencia* spp., *Salmonella* spp., *Serratia marcescens*, *Shigella boydii* and *Shigella flexneri*) from our laboratory collection.

### scFv isolation from murine hybridoma cell lines

The scFv sequences were accessed from murine hybridomas (mAb 2E11) secreting Stx2 antibodies [14]. Total RNA was extracted from 6x10^5^ cells/mL with the RNeasy Mini Kit (QIAGEN, Hilden, Germany), following the manufacturer's recommendations, and reverse transcribed using random hexamer primers supplied by First-Strand cDNA Synthesis (GE Healthcare, UK). The amplification of heavy and light chain variable domains was carried out using degenerate primers [32], and the amplicons were sequenced to confirm the sequences as variable chains. After confirmation, the synthetic gene for the scFv was designed and synthesized by GeneScript (genescript.com). The gene was amplified for cloning by the primers: V_L_ fwd (5’cct atg cat ccg att aca aag atg acg atg aca aag gcg gtg ata tcc agc tga ccc aga g 3’), V_L_ rv (5’ ctg cca cca cta cta cca cta gcg gca gta gta ccc ttc agt tct aat ttg gta cc3’), V_H_ fwd (5’gtg gta gta gtg gtg gca gta gca gtg gtg ccg aag tga aac tgg tgg aaa gcg3’), and V_H_ rv (5’ttg tcg gcc gag ctc acg gtc agg3’). All DNA/RNA gels contained a 1-kb Plus molecular ladder (Invitrogen, CA, USA).

### Selection against immobilized antigens to obtain Fab fragment

#### Fab-phage library screening

Library screening was performed using the human synthetic antibody library F displayed on the surface of M13 bacteriophage in a bivalent format [29]. Selection and panning were performed against immobilized antigens following the standard protocol as previously described [33]. Briefly, a 96-well microplate was coated with 5 μg/mL purified Stx2 toxin (100 μL/well) in phosphate buffered saline (PBS) at room temperature for 2 h, followed by blocking for 1 h with 200 μL/well PB buffer (PBS with 0.2% BSA). The plate was incubated with 100 μL/well of library phage solution (10^12^–10^13^ pfu/mL) in PBT buffer (PBS, 0.2% BSA, 0.05% Tween-20) at room temperature for 2 h with gentle shaking. The unbound phage solution was removed by washing 10 times with PT buffer (PBS, 0.05% Tween-20). Bound phage was eluted adding 100 μL/well of 100 mM HCl and incubating at room temperature for 5 min. The eluent was transferred to a 1.5-mL microfuge tube containing 1.0 M Tris-HCl, pH 11, to adjust to neutral pH. Half of eluted phage solution was added to 10 volumes of actively growing *E*. *coli* Omnimax 2 T1^R^ (OD_600_ < 1.0) in 2YT medium containing 10 μg/mL tetracycline (tet^10^), which were then incubated at 37°C for 20 min with shaking at 200 rpm. Serial dilution of phage eluted was performed to determine the enrichment ratio as described by Tonikian et al. [34]. Four rounds of panning were performed. The monoclonal phage particles were grown and tested by ELISA [18] and positive clones were then sequenced.

### Cloning

The selected phages and synthetic gene were amplified again and purified with the Qiaquick PCR purification kit (QIAGEN, Hilden, Germany) followed by digestion together with the expression vector pFabHis-MBP and pscFvHIS-MBP with restriction enzymes *Nsi*I/*Sal*I and *Eag*I/*Sal*l, respectively. The digested fragments were again purified with the same kit and quantified using a NanoDrop 2000 spectrophotometer (Thermo Scientific). Cloning was performed using 1:3 (vector/insert) ratio with T4 ligase enzyme, and the reaction mixture was incubated at room temperature for 1 h. Afterwards, the sample was transformed into prepared competent cells [35]. Cloning was confirmed either by double digestion or PCR amplification.

### scFv expression and purification

A single colony was cultivated in 30 mL 2YT carb^100^ medium at 37°C for 16–18 h at 200 rpm. Next, 20 mL of this culture were added to 1 L of 2YT carb100 medium and incubated at 37°C at 200 rpm until reaching OD_600_ 0.6, after which an aliquot of 1 mL was reserved for stocks in 75% glycerol. The culture was cooled down to 18°C for 30 min. scFv expression was induced by adding 1 mM IPTG and the culture was incubated overnight at 18°C, 200 rpm,. The culture was centrifuged at 12,042 x g for 20 min. The supernatant was discarded and the pellet stored at −20°C. The cells were lysed with 40 mL of lysis buffer (182 mL of modified PBS buffer—50 mM NaH_2_PO_4_, 300 mM NaCl, pH 8.0–4 mL of 40 mM imidazole, 40 mg lysozyme, 10 mL of 10% Triton X100, 1 mL of 2 M MgCl_2_, 1% 100X PIC, 1 μL of benzonase) and incubated at 4°C with gentle shaking for 30 min. The samples were disrupted at 20% amplitude pulses, 5 s on and 5 s off, for 2 min and cooled in ice. The lysates were centrifuged at 25,932 x g for 50 min at 4°C. The supernatant was mixed with 2 mL of 50% Ni-NTA and incubated with gentle shaking at 4°C for 1 h. The sample was centrifuged again at 106 x g for 1 min before being loaded on the column. The column was washed 3 times with 10 mL of wash buffer (147 mL of modified PBS and 2 mL of 2 M imidazole). Finally, the purified protein was eluted 3 to 5 times with 1 mL of elution buffer (20.6 mL of modified PBS and 4.4 mL of 2 M imidazole), and the fractions obtained in the process were analyzed by SDS-PAGE and quantified using a NanoDrop 2000 spectrophotometer.

### Fab fragment expression and purification

A single colony was cultivated in 15 mL of 2YT medium containing 100 μg/ml of carbenicillin and chloramphenicol (10 μg/mL; cm^10^) at 37°C and 200 rpm for 16–18 h. Ten milliliters of the culture were added to 1 L of 2YT carb100 medium and cells grown under the same conditions up to the logarithmic phase (OD_600_ 0.6–0.8). The culture was cooled at room temperature for 30 min, and an aliquot of 1 mL was taken for stocks in 75% glycerol. The expression vector was induced by the addition of 500 μL of 1 M IPTG, and the culture was incubated at 25°C and 200 rpm for 16–18 h. The cells were centrifuged at 12042 x g, at 4°C for 20 min, and the pellet was stored at −20°C for 16–18 h. The pellet was ressuspended in 40 mL of lysis buffer (45 ml of Tris buffer −12.11 g Tris and 54.44 g NaCl, pH 7.4, in 2 L of distilled water −0.5 mL PIC −3.28 g PMSF, 3.12 g benzamidine and ethanol up to 200 mL −0.25 mL Triton X100, 50 mg lysozyme, 0.25 mL of 2 M MgCl_2_, 1 μL of benzonase and 5 mL of 50% glycerol) and incubated at 4°C for one hour with gentle shaking. The sample was then heated at 65°C for 30 min and cooled on ice for 5 min. Lysed cells were pelleted by centrifugation at 25,932 x g for 30 min and the supernatant was subjected to purification. For Fab purification, 2 mL of rProtein A Sepharose Fast Flow were added to a column (Bio-Rad, CA, USA) followed by equilibration with 5 column volumes of Tris buffer, and the sample was then loaded on the column followed by washing with 10 column volumes of Tris buffer. The Fab fragments were eluted with 5 mL of 0.1 M acetic acid in microfuge tubes containing 1.5 mL of neutralization buffer (1 M Tris-HCl, pH 8.0) and mixed immediately with a micropipette. The column was equilibrated with 10 column volumes of Tris buffer and stored in 20% ethanol. The samples were quantified using a NanoDrop 2000 spectrophotometer and analyzed by SDS-PAGE using as molecular weight BLUeye Prestained Protein Ladder (Sigma-Aldrich). The buffer exchange was performed by using 10 k Amicon Ultra-4 (Millipore, MA, USA) with centrifugation at 4000 rpm for 15 min. The sample was ressuspended in 2 mL of PBS and quantified using a NanoDrop 2000 spectrophotometer.

### Interaction of recombinant antibodies with toxins: binding, definition of detection limit and affinity

The features of fragments were determined by ELISA: the binding efficiency was determined using a 384-well plate coated with 2 μg/mL antigen and incubated overnight at 4°C with gentle shaking. The plate was blocked with 60 μL/well of PB buffer for 1 h at room temperature with gentle shaking. The plate was washed 6 times with PT buffer. After blocking, a 3 log serial dilution of Fab/scFv, starting with 20 μg/mL, was performed in PBT and the plate incubated for 30 min at room temperature with gentle shaking. The plate was washed 8 times with PT. Afterwards, 30 μL/well of HRP antibody/anti-Flag conjugated to peroxidase (1:5000) in PBT were added to the plate, which was then incubated for 30 min at room temperature with gentle shaking. The plate was again washed 8 times with PT. The reaction was developed by adding 30 μL/well of TMB (1:1) and stopped by adding 30 μL/well of 1 M H_3_PO_4_. The plate was read with a 450 nm filter. The effective binding concentration was determined by calculating the antibody concentration used to reach half the absorbance curve. This concentration was applied to performed the cross-reaction ELISA using both Stx toxin (2 μg/mL) in a 96-well plate using 0.2% BSA (control); for detection limit, a 384-well plate (Maxisorp, Nunc, NY, USA) was coated with 5 μg/well antigen and incubated overnight at 4°C with gentle shaking. The plate was blocked with 60 μL/well of 0.2% PB buffer for 1 h at room temperature with gentle shaking. After blocking, Fab/scFv at the effective binding concentration was pre-incubated with 3 log dilutions of antigen starting at 100 nM in non-binding plates (Corning, NY, USA) for 1 h at room temperature with gentle shaking. The coated and blocked plate was washed 6 times with PT buffer. Afterwards, 30 μL/well of pre-incubated Fab were added to the coated plate, which was incubated for 15 min at room temperature with gentle shaking, followed by washing with PT 8 times. Next, 30 μL/well of HRP antibody/anti-Flag conjugated to peroxidase (1:5000) in PBT were added to the plate, which was then incubated for 30 min at room temperature with gentle shaking. The plate was again washed 8 times with PT. The reaction was developed by adding 30 μL/well of TMB (1:1) and stopped by adding 30 μL/well of 1 M H_3_PO_4_, and the plate was read with a 450 nm filter. The dissociation constants (*K*_*D*_) were determined as described by Friguet et al. [36], using Scatchard plots.

### Peptide mapping

Peptide mapping was performed using *CelluSpot Peptide Array* (Intavis, www.intavis.com) following the manufacturer’s recommendations. Briefly, the slides were blocked with PBS-1% BSA at room temperature for 4 h, followed by incubation with 250 μg/mL Fab or scFv in blocking solution at room temperature for 3 h. The slides were then carefully rinsed with blocking solution and washed 3 times with PBT, 5 min each. Next, the slides were incubated at room temperature for 1 h with HRP/anti-FLAG horseradish peroxidase conjugate (1:5000). After washing, DAB and H_2_O_2_ were added and the reaction was stopped after 15 min by the addition of distilled water.

### Structure analysis

We used the PyMol program (DeLano Scientific LLC, 2009) to predict the structure of the recombinant antibody and the epitope of Stx2. For the Stx2 structure, we used the available PDB file (10.2210/pdb1r4q/pdb) published by Fraser et al. [37]. For the structure of recombinant antibodies, we first performed the prediction with Phyre [38] and used the PDB file on PyMol.

### Vero cell toxin assay

Vero cells (1x10^5^ cells/mL) were grown in 96-well plates in Dulbecco’s modified Eagle medium (DMEM) in the presence of 10% FBS for 24 h for neutralization assays. Cells were also cultivated under the same conditions in 8-well plates (Chamber Slide, Nunc, NY, USA) for the immunofluorescence assay.

### Antibody neutralization assays

The neutralizing ability of Fab and scFv anti-Stx2 recombinant antibodies was determined by incubating Stx2 toxin at the CD_50_ (defined by Rocha et al. [14]) or DMEM (negative control) with the effective concentration (determined by the first step of the three-step ELISA for K_D_ determination) of Fab or ScFv at 37°C for 72 h with 5% CO_2_. Monoclonal anti-Stx2 antibody was employed as neutralizing activity control at the same concentration used by Rocha et al. [14]. After incubation, MTT (Sigma-Aldrich) was used to determine cell viability according to the manufacturer’s instructions. These assays were performed three times in duplicate.

### Sensitivity and specificity test

The reactivity with the toxin expressed by STEC isolates (sensitivity) or with the non-toxin-producing strains (specificity) was determined by capture ELISA using microplates coated with the IgG-enriched fraction of anti-Stx2 (3 μg) rabbit polyclonal serum [39,40] at 4° C for 16–18 h. After blocking with 1% BSA at 37°C for 30 min, 100 μL of isolate supernatant were incubated at 37°C for 2 h followed by incubation with 0.5 μg scFv. Antigen-antibody binding was detected by the addition of HRP/anti-FLAG horseradish peroxidase conjugate (1:5000). The reaction was developed by adding 30 μL/well TMB (1:1) and stopped by adding 30 μL/well 1 M H_3_PO_4_, and the plate was read with a 450 nm filter. scFv reactivity with Stx expressed by STEC isolates was arbitrarily defined as low (1–30 ng), medium (31–60 ng) and high (61–100 ng) compared to the absorbance obtained with the reactivity of 100 ng of purified toxins, which we considered as having a high reactivity level.

### Statistical analysis

Both ELISA and neutralization assays were analyzed by GraphPrism 5.01, using Student’s *t*-test, where the differences were considered statistically significant when p ≤ 0.05. The receiver operating characteristic (ROC) curve was employed for determining the cut-off value using the ELISA data, considering the highest sensitivity and specificity.

## Results

### scFv from mAb-IgG-secreting hybridoma

**Fig 1:**
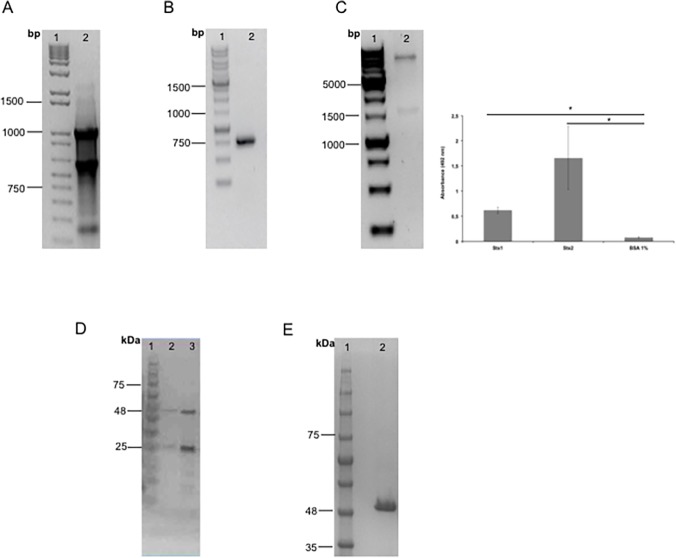
scFv and Fab purification. A. RNA extraction from Stx2 IgG-producing hybridoma. Molecular marker (lane 1); Total RNA from anti-Stx2 IgG-producing hybridoma (mAb 2E11) (lane 2). B. scFv gene cloning confirmation. Molecular marker (lane 1); PCR product (lane 2). C. scFv fragment purification. Molecular weight ladder (lane 1); scFv elutions (lanes 2 and 3). D. Phage gene cloning confirmation. Molecular marker (lane 1); Double digestion (lane 2). Phage ELISA, results of independent experiments, performed in triplicate, are expressed as the means ± SEM * p < 0.05 compared with control. E. Electrophoretic analysis of Fab fragment purification. Molecular weight ladder (lane 1); Fab fragment purified (lane 2).

The variable light and heavy chain sequences of scFv fragments were obtained from the Stx2-secreting hybridoma (mAb 2E11- IgG1, *K*_*D*_ = 6.14 x 10^–10^M) [14], where the total RNA of these cells was extracted (Fig. 1A) and retrotranscripted to cDNA followed by the amplification of the variable chains. Once amplified, the chains were confirmed as variable mouse IgG chains by sequencing and analysis of the sequences, and the synthetic gene was designed as a VL-linker-VH orientation, which turned into a non-functional scFv. We therefore decided to modify this assembly to VH-linker-VL orientation (data not shown). Therefore, PCR amplification was done and new primers were designed, and also, the GGGGSGGGGSGGGGS linker was replaced with a more flexible one, GTTAASGSSGGSSSGA. After amplification of the new assembled gene, it was cloned into an expression vector. Cloning was confirmed by PCR with the amplification of the 1000-bp scFv gene from the *E*. *coli* host cells (Fig. 1B).

### Phage-library screening for Fab

A multi-step panning against the Stx2 purified toxin was performed with a synthetic antibody library F [29]. After 4 rounds of panning, 96 phage clones were picked for monoclonal phage ELISA, and 82 clones showed positive signals against Stx2 toxin in phage ELISA, which after sequencing turned out to be only two different clones. One of them showed cross-reactivity to Stx1 and was thus chosen for further characterization. This phage clone showed, by ELISA, an absorbance at 492 nm of 0.5 and 1.5 for Stx1 and Stx2, respectively (Fig. 1C). DNA encoding the Fab fragment was amplified from the corresponding Fab-displayed phage and cloned into an expression vector. Cloning was confirmed by a ∼1500 bp insert by enzymatic digestion with *Nsi*I and *Sal*I (Fig. 1C) as well as plasmid sequencing (data not shown).

### Purification of scFv and Fab fragments

After cloning, the expression of both fragment genes was induced and the proteins were purified. Since the expressed scFv fragment had a 6X His-tag fused to the C terminus of the VH region, it was purified by affinity chromatography with a Ni-NTA column, where scFv was eluted by competition with imidazole in two steps, yielding the 25- to 30-kDa fragments (Fig. 1D, lane 3). The 50-kDa Fab fragment was purified by affinity chromatography in a protein A column (Fig. 1E). After affinity chromatography, Fab and scFv were subjected to buffer exchange and the protein concentration obtained was 1.5 and 1 mg/mL, respectively.

### Affinity and binding ability of antibody fragments to Stx2

The lowest concentration of antibody required to detect the antigen was 680 pmol for scFv and 54 pmol for Fab fragment. Following this assay, we performed the detection limit assay to determine the concentration of antigen required to saturate the paratope and block binding with the immobilized antigen. The inhibitory concentration of antigen was 10 nM for the scFv fragment and 24 nM for Fab. We also calculated the fragment apparent affinity constant: 4.9 x 10^−5^ M for scFv and 1.2 x 10^−5^ M for Fab.

**Fig 2:**
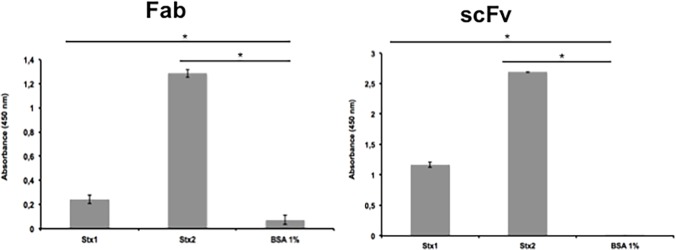
ELISA for Stx toxin detection by Fab and scFv fragments. The assay was performed in triplicate and considered positive when * p>0.05 by Student’s t-test versus control.

The binding abilities of the scFv and Fab fragments were tested by ELISA and the reactivity with both toxins was significantly higher compared to the control (p < 0.005) (Fig. 2). On the other hand, neither fragment was able to recognize the two toxins by immunofluorescence after 24 h interaction of the toxins with Vero cells (data not shown).

### Peptide mapping and structural prediction of scFv and Fab fragments

**Fig 3:**
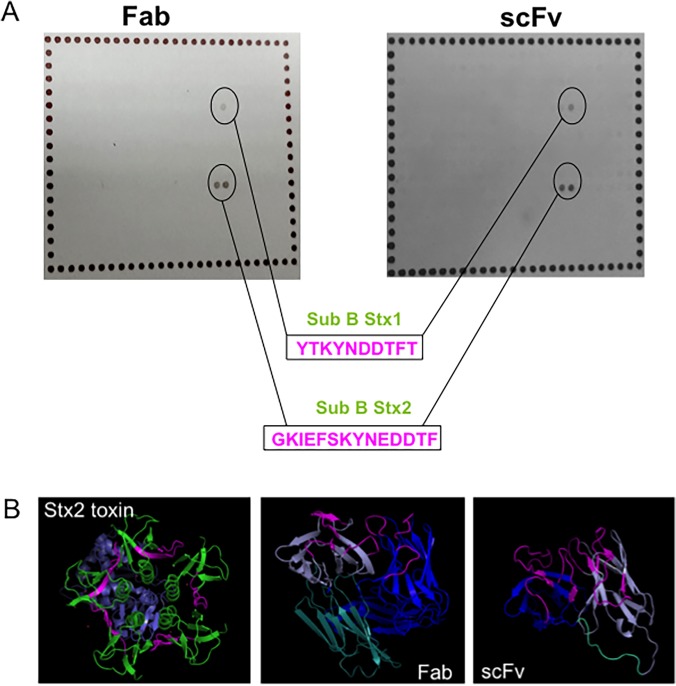
Structural analyses of antibody fragments. A. Peptide mapping and the corresponding peptides, with the epitopes highlighted in pink. B. Structure prediction of both recombinant antibodies and Stx2, subunit A (purple) and subunit B (green), with the recognized peptides highlighted in pink as well as the antibody CDRs of recombinant antibodies. Also, the variable chains are represented, heavy (blue) and light (purple). In the Fab structure, the constant chain is shown in light blue, as well as the scFv linker.

The recognition of Stx1 and Stx2 toxins by scFv and Fab prompted us to determine which epitope was involved in antibody binding. Peptide mapping showed that both fragments bound to the same epitope on the B subunit, the toxin subunit responsible for binding to the cell receptor (Fig. 3A). The peptide recognized in the Stx1 B subunit was YTKYNDDTFT (highlighted in pink, Fig. 3B), and for the Stx2 B subunit, the fragments were able to recognize the GKIEFSKYNEDDTF epitope (highlighted in pink, Fig. 3B), which is larger than the epitope in Stx1 toxin. The homologous sequence between B subunits of Stx1 and Stx2 was KYN(E)DDTF, which can explain the cross reactivity of these fragments.

The recombinant antibody structures were predicted using the Phyre program. For the scFv fragment, 242 residues (100% of the sequence) were modeled with 100% confidence using another scFv against a cytokine (c2kh2b template), and both scFv fragments showed 64% identity. For the Fab fragment, 430 residues (99% of the sequence) were modeled with 100% confidence using another Fab structure against a virus (C1qqc4 template), and both Fab fragments showed 60% identity. The predicted structures were analyzed by the PyMol program. The sequences responsible for antigen recognition (CDRs—complementary domain regions) of the fragments are highlighted in pink as well as the recognized peptide on the Stx2 toxin (Fig. 3B).

### Toxin cytotoxicity neutralizing capacity

**Fig 4:**
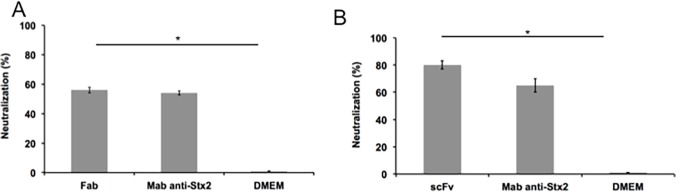
Cytotoxicity neutralization assay. (A) scFv and (B) Fab. Dulbecco’s modified Eagle medium (DMEM) was used as negative control, and anti-Stx2 mAb used as positive neutralizing control. The assay was performed in triplicate, and significant neutralization was considered when * p > 0.05 by Student’s *t*-test versus the negative control. p < 0.05 with the positive control indicated that both had the same neutralizing capacity.

The scFv fragment was able to neutralize this effect up to 80% (Fig. 4A), higher than its parental monoclonal antibody mAb 2E11 [14]. The Fab fragment selected from a human synthetic antibody library was able to neutralize up to 70% of the cytotoxic effect of Stx (Fig. 4B). Also, The differences were statistically significant when compared to the cytotoxic effect of the toxin (p < 0.005). Compared to anti-Stx2 mAb, Fab showed high neutralizing capacity (p > 0.005) (Fig. 4B).

### Sensitivity and specificity of scFv fragment

**Fig 5:**
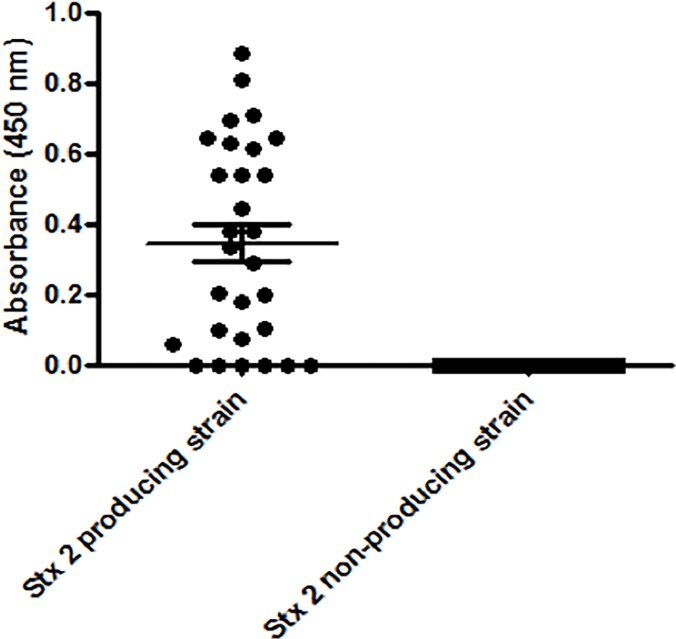
ELISA for toxin detection using supernatant of Stx-positive and—negative isolates with scFv fragments. The assay was performed in triplicate and the ROC curve determined the cut-off as 0.028.

The scFv fragment was not a human-derived fragment, and thus, to use it as a therapeutic agent, it would be necessary to humanize it. Therefore, this fragment was tested as a tool for diagnosis. Accordingly, the STEC collection and Stx-non-producing strains were evaluated by capture ELISA; the scFv fragment was able to recognize the majority of Stx2-producing strains, with 79.3% sensitivity (confidence interval of 60.3 to 92%), and no reactivity was observed with the non-producing strains, indicating as high as 100% specificity (confidence interval of 86.8 to 100%) (Fig. 5).

## Discussion

Although several monoclonal antibodies against Stx toxins have been described in the past years [3,12,13,14,15,16,17,18,19,20,21,22,24], STEC diagnosis and treatment for intoxication remain critical, thus the development of new tools such as recombinant antibodies is still required. Monovalent scFv and Fab antibodies can be produced very efficiently using bacterial protein synthesis systems, and this would be helpful in reducing time and cost, thus being an alternative to hybridoma culture [27]. Herein, two recombinant antibody fragments targeting Stx2 were development and characterized.

scFv is the format where the V_H_ and V_L_ are joined by a flexible polypeptide linker to prevent their dissociation. Besides the variable chains, Fab antibodies also have one constant region in each chain. Both fragments retain the specific, monovalent, antigen-binding affinity of the parent IgG, while showing improved pharmacokinetics for tissue penetration [25]. In a previous work, it was established that anti-Stx2 mAb showed high sensitivity in detecting Stx by capture ELISA, even in low-producing isolates, besides exhibiting *in vitro* neutralizing capacity [14], resulting in a rebuilt scFv fragment.

The scFv antibody fragment constructed here was obtained from a bacteria-induced culture and showed a diagnostic ability, since it was able to detect the STEC strains by ELISA. None of the commercially available immunoenzymatic tests for Stx1/2 toxin detection [41] employ recombinant antibodies produced in bacteria. Additionally, the scFv fragment described here was able to block toxin cytotoxicity *in vitro* up to 80%, which was even better than its precursor IgG molecule. Other recombinant antibodies have also shown neutralizing ability against Stx2 [23,28,42]. The VHH and the scFv recombinant fragments developed by Tremblay et al. [23] and Maa et al. [42], respectively, also protected mice against Stx2 intoxication, but none of them is a humanized antibody, which impairs their use as therapeutic agents. On the other hand, the human Fab and F(ab’)2 fragments evaluated by Akiyoshi et al. [28] showed neutralizing capacity, but since they were produced in intestinal cells (CHO), there was still the problem of high cost as with hybridoma technology. Considering this information, and since the scFv produced herein showed *in vitro* neutralizing ability, we made an effort to obtain a therapeutic human antibody expressed in bacteria through the selection of clones from a synthetic antibody library displayed on phage surfaces which compete for the same epitope as the scFv previously tested. The Fab clone identified from library F in this work showed epitope competition with both scFv and IgG antibodies (data not shown), indeed binding to the same peptide.

Both engineered antibodies bound to the same epitope on subunit B of Stx2, which showed some conserved residues with the Stx1 B subunit, leading both fragments to cross-react with both toxins by ELISA. This epitope is displayed on the B pentamer and is an important epitope for the toxicity of the toxin, since the neutralization assay with both fragments showed a higher ability to neutralize the purified antigen compared to the monoclonal antibody described by Rocha et al. [14]. This neutralizing capacity seems to involve blocking of the receptor binding site, preventing the translocation of effective subunit A into the target cells. Other neutralizing antibodies and recombinant antibody fragments have also shown specificity for subunit B [19,23,43,44,45], where indeed, the majority of Stx2 antibodies react with subunit B. The recombinant antibodies obtained herein can bind to each of the five B-subunits of the holotoxin, which can improve the neutralization efficacy as shown by the anti-Stx2 mAb described by Cheng et al. [43]. The same occurred with TMA-15, which despite providing mouse protection after 24h of infection, is produced in mammalian cells [44].

Differences in affinity constants of mono- or dimeric antibodies are due to changes in avidity [46]. The affinity constants of rAbs are usually lower than those of IgG as shown by both scFv and Fab described in the current work and the scFv-Fc developed against botulinum toxin [47]. In the case of the Fab fragment, it can be converted into an IgG4 human molecule by subcloning, which may enhance antigen binding since it will be a more stable dimeric molecule. This work is currently underway in our laboratory.

In conclusion, in the present work, we successfully developed and characterized two different recombinant antibody types, mouse-scFv and human-Fab fragments, achieved by different approaches, i.e., hybridoma and phage display, respectively, and for the first time, we described a human monovalent molecule, produced in bacteria, capable of neutralizing the cytotoxicity of Stx2 *in vitro*. Thus, these rAbs can be used as diagnostic and therapeutic tools in dealing with Stx2 toxin.
